# Pupillometry as a new window to player fatigue? A glimpse inside the eyes of a Euro Cup Women’s Basketball team

**DOI:** 10.5114/biolsport.2024.125590

**Published:** 2023-05-25

**Authors:** Thomas Huyghe, Julio Calleja-González, Stephen P. Bird, Pedro E. Alcaraz

**Affiliations:** 1 Research Center for High Performance Sport, Universidad Católica de Murcia, Murcia, Spain; 2 Department of Physical Education and Sports, Faculty of Education and Sport, University of the Basque Country, (UPV/EHU), Vitoria-Gasteiz, Spain; 3 Faculty of Kinesiology, University of Zagreb, Zagreb, Croatia; 4 School of Health and Medical Sciences, University of Southern Queensland, Ipswich QLD, Australia

**Keywords:** Eye-tracking, Neurotechnology, Neuroimaging, AMS, Athlete monitoring

## Abstract

A rapidly emerging area of interest in high-pressure environments is that of pupillometry, where handheld quantitative infrared pupillometers (HQIPs) are able to track psycho-physiological fatigue in a fast, objective, valid, reliable, and non-invasive manner. However, the application of HQIPs in the context of athlete monitoring is yet to be determined. Therefore, the main aim of this pilot study was to examine the potential usefulness of a HQIP to monitor game-induced fatigue inside a professional female basketball setting by determining its (1) test-retest repeatability, (2) relationship with other biomarkers of game-induced fatigue, and (3) time-course from rested to fatigued states. A non-ophthalmologic practitioner performed a standardized Pupil Light Reflex (PLR) test using a medically graded HQIP among 9 professional female basketball players (2020–2021 Euro Cup) at baseline, 24-h pre-game (GD-1), 24-h post-game (GD+1) and 48-h post-game (GD+2). This was repeated over four subsequent games, equalling a total of 351 observations per eye. Two out of seven pupillometrics displayed good ICCs (0.95–0.99) (MinD and MaxD). Strong significant relationships were found between MaxD, MinD, and all registered biomarkers of game-induced fatigue (r = 0.69–0.82, p < 0.05), as well as between CV, MCV, and cognitive, lower-extremity muscle, and physiological fatigue markers (r = 0.74–0.76, p < 0.05). Three pupillometrics were able to detect a significant difference between rested and fatigued states. In particular, PC (right) (F = 5.173, *η*^2^ = 0.115 p = 0.028) and MCV (right) (F = 3.976, *η*^2^ = 0.090 p = 0.049) significantly decreased from baseline to GD+2, and LAT (left) (F = 4.023, *η*^2^ = 0.109 p = 0.009) significantly increased from GD-1 to GD+2. HQIPs have opened a new window of opportunity for monitoring game-induced fatigue in professional female basketball players. However, future research initiatives across larger and heterogenous samples, and longer investigation periods, are required to expand upon these preliminary findings.

## INTRODUCTION

In high-performance sports, excessive levels of fatigue can inhibit the desired adaption to training, increase injury risk, and potentially hinder athletic performance [1]. Therefore, continuously exploring new ways to quantify player readiness is considered a priority within elite sporting organizations [1, 2]. In light of this pursuit, numerous fatigue monitoring tools have emerged [1, 2]. However, from a practical perspective, traditional fatigue monitoring tools often remain exhaustive (e.g., maximal-effort physical testing) [2, 3], subjective (e.g., self-reported questionnaires) [2, 4], invasive (e.g., blood sampling) [2, 5], expensive (e.g., electroencephalogram) [2, 6], or relatively slow to conduct (e.g., 5-min recordings of heart rate indices in standing and lying postures) [7]. Hence, there’s an ongoing need for innovative solutions that enable real-time, multi-modal, non-invasive, cost-effective, valid, and reliable insights into player fatigue, and in turn, improve the day-to-day decision-making processes of coaches and support staff personnel [1, 2].

Some of the most promising innovations to date in this space have emerged from collaborative initiatives between engineers, developers, scientists, and practitioners who operate in high-pressure environments (i.e., transatlantic flights, space shuttle missions, military combat, medical surgery, long-haul truck driving, etc.) as a lack of operational readiness in these positions could lead to lethal consequences [8, 9, 10]. Consequently, pupillometry has gained a rapid surge in interest by the research community across high-stake industries [9, 10]. Pupillometry can be defined as the study of the the central opening of the iris through which light passes before reaching the lens and being focused onto the retina [11]. Because the pupils are directly innervated by the second cranial nerve (CN II) and third cranial nerve (CN III) [11], measuring pupil reflexes provides an objective representation of the autonomic nervous system (ANS) [12–15] as well as cognitive, emotional, physical, and physiological status in real time [16, 17, 18]. Since the first discovery of pupillometry as a human fatigue detection tool in 1936 [19], the field has rapidly advanced in recent years due to the emergence of Handheld Quantitative Infrared Pupillometers (HQIPs) [19, 20, 21, 22]. In particular, HQIPs are now able to repeatedly measure the pupil diameter (1 measurement every 30 ms) with a minimum detectable change of < 0.03 mm (i.e., practical error of 0.88% in relation to the average pupil diameter) [22, 23]. Consequently, a vast range of Intensive Care Units (ICUs) settings [24] and high-stake occupations are progressively integrating HQIPs as a first-point-of-care instrument [25, 26, 27].

Surprisingly, the use of modern HQIPs in professional sports remains bounded by a few use cases (e.g., concussion-related diagnostics [28, 29, 30] and “quiet eye” analytics [31]). While some researchers have introduced HQIPs as a method to evaluate ANS function in athletes [12, 14, 15], the validity and reproducibility of their methods and findings remains unclear. For instance, the investigations typically followed a cross-sectional study design, adopted non-standardized and non-validated pupil testing procedures, executed in laboratory conditions, and involved only amateur and subelite athletes. Besides the application of HQIPs to monitor ANS function, researchers have also demonstrated its effectiveness to monitor cognitive effort (i.e., pupil dilation can be viewed as an indirect index of effort in cognitive control tasks across the domains of updating, switching and inhibition) [32]. This could imply an important discovery as player performance and fatigue originates from the complex state of both physiological and psychological processes [33]. Hence, HQIPs may potentially reveal itself as a multi-model at monitoring instrument.

Acknowledging the inherent potential of HQIPs, and appreciating the efforts made by previous researchers on this research line, this pilot study aims to explore the potential usefulness of a medically graded HQIP to monitor game-induced fatigue in nine professional female basketball players by determining (1) the test-retest repeatability, (2) the relationship between pupillometrics and other biomarkers of game-induced fatigue, and (3) the time-course of pupillometrics from baseline and 24 h before games up to 24 h and 48 h following games. In turn, the reported baseline findings and methodological framework may serve as a valuable reference for future research initiatives on this topic.

## MATERIALS AND METHODS

### Experimental approach to the problem

This pilot study followed a prospective observational study design and was conducted in non-experimental conditions, so the coaching staff, support staff personnel, and participants did not receive any input from the research team. Training data, competitive schedule and fixture outcomes were supplied by the coaching staff of the team. Two weeks prior to the investigation period, a baseline pupil test was performed after two consecutive off days (i.e., no scheduled or organized practices or workouts during these days) to optimize physical and psychological recovery. Subsequently, the participants played four home games over a 5-week investigation period (1 week apart, all games commenced between 8:00 – 8:30 PM). For each game, a pupil testing sequence was executed at the following timepoints: 24-h pre-game (GD-1), 24-h post-game (GD+1), and 48-h postgame (GD+2). All pupil tests were completed and performed inside a standard clinical testing room during regular pre-practice physiotherapy session hours (6:00 PM – 7:30 PM) to emulate a standardized clinical testing time and environment.

### Participants

Nine female Belgian professional basketball players (n = 9) competed in the 2020–2021 Euro Cup Women’s Basketball Tournament and voluntarily participated in this study. All participants were aged 18 years or older (range: 18–33 years; mean age: 21.20±4.92 years), with a mean height of 181 ± 5.36 (cm) and body mass of 80.61 ± 10.73 (kg). Based on positional groupings: 45% were grouped as Posts, 33% as Guards, and 22% as Wings. Based on the role: 55% were starters and 45% non-starters.

Players were not eligible to participate when they encountered at least one of the following criteria: < 18 years of age; unable to participate in individual and/or team practices due to injury or illness at any point of the investigation period; unable to sit for testing; history in genetic syndromes, neurologic pathologies (including intracranial masses) or intraocular pathologies that would affect pupillary function (e.g. uveitis, cataracts, diabetes, glaucoma, optic nerve dysfunction); ingestion of alcoholic and/or caffeinated foods, drinks, or substances within < 12 h of any pupil examinations; use of ergogenic aids and/or medical support that may have altered the neurophysiological state of the athlete at any point of the investigation period. Prior to the investigation, this study was approved by the Institutional Review Board of UCAM University, Murcia, Spain (code: CE122002) and conformed to the requirements of the European Union General Data Protection Regulation and United States Health Insurance Portability and Privacy Act with adherence to the tenets of the Declaration of Helsinki with Fortaleza actualization 2013 [34]. All test procedures strictly adhered to the World Health Organization (WHO), European Commission, and local government safety guidelines regarding scientific research during the COVID-19 pandemic.

### Testing procedure

To verify whether any pupillometrics could detect a significant change in game-induced fatigue and recovery, participants were instructed to go through a comprehensive fatigue test battery at each allocated timepoint (i.e., baseline, GD-1, GD+1, GD+2). The fatigue test battery consisted of the pupil test in combination with four other fatigue tests: cognitive fatigue test (i.e., visuomotor reaction time) [35, 36], lower-extremity muscle fatigue test (standing postural sway) [37, 38], perceptual fatigue test (self-perceived exertion) [38], and ANS fatigue test (heart rate variability indices) [40–44]. More specifically, upon arrival to the clinical testing room, the player was instructed to wear the Polar H10 heart rate chest strap (Polar

Electro Oy, Kempele, Finland) and complete a 5-min heart rate variability (HRV) test in rested condition and seated posture using the EliteHRV software (Asheville, NC, United States) [44] on an iPhone SE (Apple Inc., Los Altos, California, United States). The Polar H10 was selected based on its underlying support as a medically graded heart rate sensor [40, 41] and the EliteHRV was selected based on its ability to record, store, and export HRV data in a secure and user-friendly manner [44]. Particularly, the natural log of the root-mean-square difference of successive normal RR intervals (ln-RMSSD) was used for HRV analyses given its well-documented support for monitoring physiological fatigue in young female basketball players [41] as well as numerous other sport athletes [43]. Following the HRV test, the player completed two subsequent Sway tests using the Sway Medical, Inc. software (Tulsa, Oklahoma, United States) [35–38] via touch screen display as well as tri-axial accelerometry (i.e., motion detection) on an iPad (6^th^ generation) by Apple Inc. (Los Altos, California, United States). The Sway test protocols have been established as an objective and reliable method for assessing reaction time, impulse control, timed visual processing, and working memory [35–38]. Particularly, the first Sway test examined the cognitive fatigue status through the Simple Reaction Time (SRT) test (ms) [35]. During this test, the player held the iPad horizontally (landscape mode) and moved it as fast possible in any direction when the screen display changed from a white to orange color. Each SRT test started after a variable delay of 2–4 s in order to prevent the player from anticipating the stimulus ahead of time. Each player completed five trials. The fastest and the slowest SRT scores were excluded in order to remove outliers and reflect only the typical response times of the player [34]. Subsequently, the scores of the three remaining trials were averaged to calculate the individual score for each player. Following the SRT test, the player performed the Sway Balance test, which quantified postural sway during the performance of a series of tasks to reflect lower-extremity muscle fatigue [45]. Specifically, the Sway Balance test consisted of five stance conditions (10-s in duration per stance) on a firm surface and with the eyes closed. The postural sway was quantified through the iPad’s triaxial accelerometer, and the units that corresponded with the accelerations were used to calculate the final proprietary Sway Balance score [38].

Subsequently, the test administrator manually performed the standard Pupil Light Reflex (PLR) test [12, 28] in each player’s eye respectively, using the NeurOptics NPi-200 pupillometer (NeurOptics, Laguna Hills, CA, U.S.A.), a medically graded HQIP (Class I medical device as listed under 21CFR 886.1700) [11, 46]. More specifically, this HQIP integrated a calibrated full-field white light stimulus with peak wavelengths comprised of red, green, and blue at a fixed intensity (1000 Lux) and fixed flash duration (0.8 s) to simulate a standard pupil light reflex (PLR) [11, 46]. Subsequently, this HQIP digitally registered the pupil light response as a video (sampling rate of 30 Hz) for a duration of 3.5 s, followed by a display of numeric results on a screen for each eye respectively [11, 46]. The device highlighted an outline of the pupil and graphed its displacement over time with an accuracy of 0.03 mm (i.e., practical error of 0.88% in relation to the average pupil diameter) [11, 46]. Scotopic lighting conditions (434–440 lumen/m^2^) were verified prior to each pupil exam by measurement of luminance of less than 2 Lumens with a luminometer (Dr. Meter LX1330B Digital Illuminance/Light Meter, Hisgadget, Union City, CA, U.S.A.) at the level of the players’ eyes. Furthermore, normal forehead temperature was measured and controlled (35.4 °C to 37.4 °C) prior to each test via a forehead thermometer (iProven DMT-489, Beaverton, Oregon, U.S.A.). Each pupil test was conducted sitting stationary looking straight ahead. Each player was prompted to maintain a forward head posture and binocular viewing conditions in a seated position throughout the test. The non-test eye was fixated on a neutral wall at 3-m distance to the chair’s front leg. The right eye was tested first, immediately followed by the left eye. This sequence was completed three consecutive times using 60-s intervals to allow sufficient recovery of the pupil before the next light stimulus [11, 46, 47]. A retest was taken whenever the HQIP was held incorrectly, or blinking was detected by the HQIP. All pupil tests were relatively quick to conduct and did not exceed ~4 min in duration per player, and ~60 min in total duration for the entire team. Notably, ease of use was reported by the test administrator (i.e., performance coach without previous clinical experience in using HQIPs). In particular, a total of 351 pupillary measurements were recorded in each eye, without any interference with the daily predetermined schedule of the team.

The selected HQIP extracted seven pupillometrics, which represented parameters of both the Sympathetic Nervous System (SNS) function and Parasympathetic Nervous System (PNS) function [11]. Furthermore, the HQIP used an algorithm to calculate the overall reactivity of the pupil (proprietary score), called the Neurological Pupil Index (NPi) [11]. However, the authors excluded the NPi pupillometric from the final analyses as the company did not publicly provide any details on the computation of the NPi. Descriptions and calculations for the seven remaining pupillometrics are presented in Table 1.

**TABLE 1:** Descriptions of All Pupillometrics.

	Pupillometrics	Units	Description
**MaxD**	Maximum Diameter	Mm	Maximum pupil size before constriction.

**MinD**	Minimum Diameter	Mm	Pupil diameter at peak constriction.

**PC**	Percentage of Change	%	The change in pupil size over time, computed as: $PC=\left(\frac{MaxD-MinD}{MaxD}\right)*100$

**LAT**	Latency	mm/s	Time of onset of constriction following initiation of the light stimulus.

**CV**	Constriction Velocity	mm/s	Average of how fast the pupil is constricting after exposure to light.

**MCV**	Maximum Constriction Velocity	mm/s	Represents the maximum velocity of pupil constriction.

**DV**	Dilation Velocity	mm/s	The average pupillary velocity when, after having reached the peak constriction, the pupil tends to recover and dilate back to the initial resting size.

Finally, within < 1 h following any practice or game, the players completed an online survey to record their RPE score based on Borg’s rate of perceived recovery status scale of 100 points [38], in which 0 means ‘very poorly recovered/extremely tired,’ 20 represents ‘poorly recovered/very tired,’ 40 means ‘minimally recovered/tired,’ 50 denotes ‘slightly recovered/somewhat tired,’ 60 signifies ‘moderately recovered,’ 80 represents ‘well recovered,’ and 100 represents ‘very well recovered/highly energetic’ [39].

### Statistical Methods

Prior to the statistical analyses, normal distribution of the dataset was confirmed (Shapiro-Wilkinson test; n > 50). Participant demographic information, including: age, height, body mass, playing position and role were calculated using descriptive statistics. The pupillometrics were compared between the left and the right eye through a paired t-test. The intraclass correlation coefficients (ICCs) were computed to examine test-retest reliability for each pupillometric using the thresholds outlined by Martins et al. (2014) for the assessment of technological equipment in research and clinical practice: very poor: ICC < 0.70, poor: ICC = 0.70–0.90, moderate: ICC = 0.90–0.95, good: ICC = 0.95–0.99, and very good: ICC > 0.99 [48]. The Pearson’s Product Moment Correlation (r) examined the linear relationship between each pupillometric and various other measures of game-induced fatigue and recovery, including: perceptual fatigue (i.e., average daily Borg Rating of Perceived Exertion scores) [39], lower-extremity muscle fatigue (i.e., Sway Balance Error Scoring System test scores) [45]; cognitive fatigue (i.e., Sway reaction time score) [34], and ANS fatigue (i.e., lnRMSSD) [42]. The Pearson’s correlation coefficients were interpreted using the reference standards by Hopkins et al. (2009): trivial: r < 0.1; small: 0.1 < r < 0.3; moderate: 0.3 < r < 0.5; large: 0.5 < r < 0.7; very large: 0.7 < r < 0.9; nearly perfect: r > 0.9; perfect: r = 1 [49, 50]. To explore whether any pupillometrics differed between rested conditions (baseline and GD-1) and fatigued conditions (GD+1 and GD+2) at the group level, the Levene test was applied as a derivation of the classical one-way analysis of the variance (ANOVA) to compute the F-statistics, Effects sizes (expressed as “η^2”^ or Eta Squared), Coefficient of Variation (CV), absolute and relative differences, Confidence Intervals at 95% (CI95), and p-values. The post-hoc Tukey test was examined for pairwise comparisons. The η^2^ was interpreted with the following thresholds: small effect: η^2^ = 0.01; medium effect: η^2^ = 0.06; large effect: η2 = 0.14 [49, 50]. Additionally, the magnitude of these differences were visually presented by a ‘percentage difference’ in which postgame data (value) was subtracted by either baseline data or pregame data (value) represents, and divided by the baseline or pregame data (value). The significance of all inferential statistics was set for *p* < 0.05. All analyses were performed at 95%-Confidence Interval. All statistical tests were performed using IBM SPSS Version 28.0.0.0.

## RESULTS

### Descriptive statistics

A paired sample t-test revealed statistically significant difference between left and right eye pupillometrics at the group level (mean difference = -0.034; *p*-value < 0.001). Therefore, all statistical tests and analyses were performed and analyzed for each eye separately. The normative data (means and standard deviations) of all pupillometrics (at the group level) of both eyes are displayed in Table 2a and Table 2b.

**TABLE 2A:** Descriptive statistics of all pupillometrics (right eye).

		N	Mean	Std. Deviation	Std. Error	95% CI		Min	Max

						Lower Bound	Upper Bound		
**MaxD (right)**	GD-1	35	6.3223	1.02479	.17322	5.9703	6.6743	4.01	8.11
	GD+1	35	6.3500	1.01662	.17184	6.0008	6.6992	3.97	7.91
	GD+2	34	6.3224	1.06745	.18307	5.9499	6.6948	4.16	8.22
	Baseline	8	6.4775	1.06054	.37496	5.5909	7.3641	4.63	7.97
	Total	112	6.3421	1.02446	.09680	6.1502	6.5339	3.97	8.22

**MinD (right)**	GD-1	35	3.9794	.76203	.12881	3.7177	4.2412	2.58	5.85
	GD+1	35	3.9837	.69930	.11820	3.7435	4.2239	2.58	5.23
	GD+2	34	4.0256	.73358	.12581	3.7696	4.2815	2.62	5.65
	Baseline	8	3.8788	.76868	.27177	3.2361	4.5214	2.74	5.38
	Total	112	3.9876	.72542	.06855	3.8518	4.1234	2.58	5.85

**PC (right)**	GD-1	35	.3720	.03437	.00581	.3602	.3838	.28	.44
	GD+1	35	.3769	.03151	.00533	.3660	.3877	.32	.44
	GD+2	34	.3703	.03389	.00581	.3585	.3821	.27	.42
	Baseline	8	.4013	.03796	.01342	.3695	.4330	.32	.43
	Total	112	.3751	.03404	.00322	.3687	.3815	.27	.44

**CV (right)**	GD-1	35	3.2737	.46457	.07853	3.1141	3.4333	2.38	4.37
	GD+1	35	3.3029	.42080	.07113	3.1583	3.4474	2.37	4.23
	GD+2	34	3.2750	.45240	.07759	3.1171	3.4329	2.42	4.13
	Baseline	8	3.4250	.46605	.16477	3.0354	3.8146	2.65	4.08
	Total	112	3.2940	.44317	.04188	3.2110	3.3770	2.37	4.37

**MCV (right)**	GD-1	35	5.3266	.77629	.13122	5.0599	5.5932	3.49	6.52
	GD+1	35	5.1871	1.10929	.18750	4.8061	5.5682	.63	7.04
	GD+2	34	5.2035	.66672	.11434	4.9709	5.4362	4.02	6.37
	Baseline	8	5.7250	.66002	.23335	5.1732	6.2768	4.85	6.61
	Total	112	5.2741	.86056	.08132	5.1130	5.4352	.63	7.04

**LAT (right)**	GD-1	35	.2131	.02898	.00490	.2032	.2231	.17	.30
	GD+1	35	.2223	.02787	.00471	.2127	.2319	.17	.27
	GD+2	34	.2147	.02135	.00366	.2073	.2222	.17	.27
	Baseline	8	.2150	.01604	.00567	.2016	.2284	.20	.23
	Total	112	.2166	.02573	.00243	.2118	.2214	.17	.30

**DV (right)**	GD-1	31	1.4132	.25639	.04605	1.3192	1.5073	1.02	2.28
	GD+1	34	1.3756	.20289	.03480	1.3048	1.4464	.90	1.82
	GD+2	32	1.3850	.24336	.04302	1.2973	1.4727	.97	2.14
	Baseline	7	1.4343	.24845	.09391	1.2045	1.6641	1.18	1.84
	Total	104	1.3937	.23263	.02281	1.3484	1.4389	.90	2.28

**TABLE 2B:** Descriptive statistics of all pupillometrics (left eye).

		N	Mean	Std. Deviation	Std. Error	95% CI		Min	Max

						Lower Bound	Upper Bound		
**MaxD (left)**	GD-1	35	6.0817	.99069	.16746	5.7414	6.4220	3.49	7.68
	GD+1	35	6.0891	.95812	.16195	5.7600	6.4183	3.65	7.56
	GD+2	34	6.1238	.97442	.16711	5.7838	6.4638	3.94	7.85
	Baseline	8	6.2650	1.03907	.36737	5.3963	7.1337	4.39	7.73
	Total	112	6.1099	.96662	.09134	5.9289	6.2909	3.49	7.85

**MinD (left)**	GD-1	35	3.7314	.64574	.10915	3.5096	3.9532	2.34	5.21
	GD+1	35	3.6911	.60097	.10158	3.4847	3.8976	2.45	4.92
	GD+2	34	3.7662	.63090	.10820	3.5460	3.9863	2.48	5.20
	Baseline	8	3.7687	.66827	.23627	3.2101	4.3274	2.77	4.95
	Total	112	3.7321	.62115	.05869	3.6157	3.8484	2.34	5.21

**PC (left)**	GD-1	35	.3851	.03568	.00603	.3729	.3974	.30	.44
	GD+1	35	.3929	.03259	.00551	.3817	.4041	.32	.47
	GD+2	34	.3847	.02339	.00401	.3765	.3929	.34	.44
	Baseline	8	.3975	.02964	.01048	.3727	.4223	.36	.44
	Total	112	.3883	.03087	.00292	.3825	.3941	.30	.47

**CV (left)**	GD-1	35	3.3491	.56844	.09608	3.1539	3.5444	1.60	4.21
	GD+1	35	3.2971	.45486	.07689	3.1409	3.4534	2.18	4.16
	GD+2	34	3.3165	.46990	.08059	3.1525	3.4804	2.17	4.32
	Baseline	8	3.4075	.56835	.20094	2.9323	3.8827	2.23	3.96
	Total	112	3.3271	.49930	.04718	3.2337	3.4206	1.60	4.32

**MCV (left)**	GD-1	35	5.4780	.81903	.13844	5.1967	5.7593	3.20	6.67
	GD+1	35	5.3737	.77775	.13146	5.1065	5.6409	3.45	6.77
	GD+2	34	5.3509	.73337	.12577	5.0950	5.6068	3.64	6.91
	Baseline	8	5.6800	1.02745	.36326	4.8210	6.5390	3.94	7.18
	Total	112	5.4213	.79076	.07472	5.2732	5.5693	3.20	7.18

**LAT (left)**	GD-1	35	.2320	.02753	.00465	.2225	.2415	.20	.27
	GD+1	35	.2186	.02992	.00506	.2083	.2288	.17	.27
	GD+2	34	.2118	.02167	.00372	.2042	.2193	.17	.27
	Baseline	8	.2063	.03420	.01209	.1777	.2348	.13	.23
	Total	112	.2198	.02828	.00267	.2145	.2251	.13	.27

**DV (left)**	GD-1	34	1.3765	.24277	.04164	1.2918	1.4612	.96	1.84
	GD+1	33	1.3009	.21842	.03802	1.2235	1.3784	.87	1.79
	GD+2	33	1.3936	.24903	.04335	1.3053	1.4819	.94	2.09
	Baseline	7	1.5057	.43412	.16408	1.1042	1.9072	.82	2.04
	Total	107	1.3669	.25499	.02465	1.3180	1.4158	.82	2.09

### Test-retest repeatability

Table 3 displays the ICC’s of all pupillometrics, which range from very poor to good (0.286 to 0.963). Particularly, LAT, DV, and MCV showed very poor ICCs (< 0.70), whereas CV and PC showed poor ICCs (0.70–0.90). However, MinD (left eye), and MaxD (both eyes) showed good ICCs (0.95–0.99). Minimal measurement bias was detected for all pupillometrics with the maximum bias for the left eye being +2.9% (MaxD) and right eye being +1.98% (MaxD). The average bias across all pupillometrics was 0.001 ± 0.450. When comparing baseline (BL) to post-game (GD+1 and GD+2) timepoints, the smallest read difference (SRD) was widest for MaxD (R = 0.340; L = 0.318) and MCV (R = 0.304; L = 0.263), and least for LAT (R = 0.005; L = 0.005) and DV (R = 0.074; L = 0.085). When comparing pre-game (GD-1) to post-game (GD+1 and GD+2) timepoints, the SRD was widest for MaxD (R = 0.285; L = 0.266) and MCV (R = 0.249; L = 0.199) and least for LAT (R = 0.007; L = 0.007) and DV (R = 0.066; L = 0.068).

**TABLE 3:** ICC scores for all 7 pupillometrics

Pupillometrics	ICCs (CI_95_)	

	Right	Left
**MaxD (mm)**	0.955 (0.937–0.968)**	0.963 (0.949–0.974)**
**MinD (mm)**	0.945 (0.920–0.962)**	0.955 (0.935–0.970)**
**PC (%)**	0.756 (0.680–0.819)**	0.749 (0.674–0.813)**
**CV (mm/sec)**	0.755 (0.679–0.818)**	0.827 (0.770–0.873)**
**MCV (mm/sec)**	0.626 (0.528–0.714)**	0.667 (0.575–0.748)**
**LAT (sec)**	0.452 (0.335–0.566)**	0.287 (0.165–0.413)**
**DV (mm/sec)**	0.501 (0.379–0.616)**	0.656 (0.558–0.742)**

** p < 0.001

### Relationships with other biomarkers of game-induced fatigue

With regards to perceptual fatigue, the findings demonstrated a very large positive significant correlation between average RPE and MinD (r = 0.78, p < 0.05) and MaxD (r = 0.77, p < 0.05). With regards to lower-extremity muscle fatigue, Sway Balance (left and right) showed a very large positive significant association with MaxD, MinD, CV, and MCV (r = 0.75–0.78, p < 0.05). With regards to cognitive fatigue, a large significant positive relationship was identified between Sway SRT scores and MinD (r = 0.69, p > 0.05) and a very large significant positive relationship between Sway SRT scores and MaxD (r = 0.70, p > 0.05). Finally, with regards to physiological fatigue, a very large positive significant relationship was detected between lnRMSSD scores and MinD (r = 0.77, p < 0.05), CV (r = 0.74, p < 0.05), and MCV (r = 0.74, p < 0.05) whereas a very large inverse significant relationship was found between MaxD and ln-RMSSD (r = -0.82, p < 0.05) (Table 4). All significant correlations have been highlighted in bold in table 4. Overall, the combination of MaxD, MinD, CV and MCV demonstrated to be the most representative of overall game-induced fatigue.

**TABLE 4:** Pearson’s correlation coefficients between the 7 pupillometrics and other biomarkers of game-induced fatigue and recovery.

Pupillometrics	Sway SRT	lnRMSSD	Sway Balance (Right)	Sway Balance (Left)	Average RPE
**MaxD**	0.70*	-0.82*	0.77*	0.79*	0.77*
**MinD**	0.69*	0.77*	0.78*	0.78*	0.78*
**PC**	-0.17	0.22	-0.28	-0.20	0.28
**CV**	-0.62	0.74*	-0.75*	-0.75*	0.45
**MCV**	-0.62	0.74*	-0.75*	-0.76*	0.44
**Lat**	0.14	-0.22	-0.10	-0.10	0.10
**DV**	-0.20	0.22	-0.10	0.00	0.24

* Coefficients presented in bold are significant (p < 0.05)

### Time course of pupillometrics following games (at the group level)

**FIG. 1:**
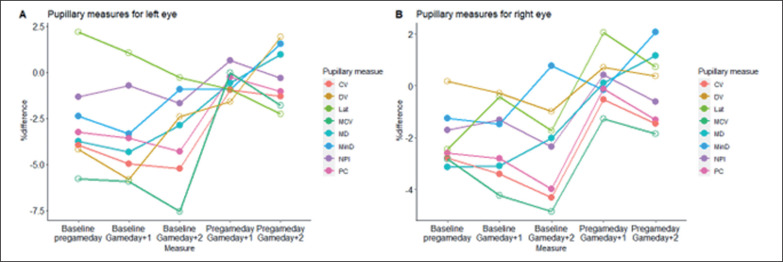
The percentage difference of pupillometrics between test moments.

Initially, the ANOVA analysis revealed that there was no statistically significant difference in pupillometrics between rested states (baseline and GD-1) and fatigued states (GD+1, GD+2) (p < 0.05), except for LAT (left) in which a medium-to-large difference was detected (F = 4.023, η^2^ = 0.109 p = 0.009). In particular, a post-hoc Tukey HSD test revealed that LAT (left) on GD-1 (0.232 ± 0.027 mm/s) was significantly higher than on GD+2 (0.212 ± 0.216 mm/s) (mean difference = 0.202, std. error = 0.006, p = 0.013, η^2^ = 0.101), thus the time from onset of the light stimulus to pupil constriction in the left eye typically took longer on GD-1 than on GD+2. Although LAT (left) was the only pupillometric that could detect a statistically significant change between rested conditions and fatigued conditions (p < 0.05), small-to-moderate effect sizes were detected for PC (right) (η^2^ = 0.052, p = 0.121), MCV (right) (η^2^ = 0.026, p = 0.410), LAT (right) (η^2^ = 0.023, p = 0.470), PC (left) (η^2^ = 0.021, p = 0.518), and MCV (left) (η^2^ = 0.013, p = 0.587). All other pupillometrics showed very small (η^2^ < 0.01) and non-significant effects (p > 0.05) across all timepoints. With regards to the magnitude of change between timepoints (% difference using Equation 1), the largest differences were found between baseline and GD+2, in which MCV (both eyes) represented the largest relative difference (left = -7.77%; right = -5.64%) (Table 5a and 5b; Figure 1).

**TABLE 5A:** ANOVA results of the pupillometric changes between baseline (BL) and post-game timepoints (GD+1 and GD+2)

ANOVA results	BL to GD+1					BL to GD+2				

	*Mean Difference*	*Std. Error*	*F*	η^2^	*p*	*Mean Difference*	*Std. Error*	*F*	η^2^	*p*
**MaxD (mm) (R)**	.127	.406	.101	.002	.752	.155	.407	.137	.003	.713
**MinD (mm) (R)**	-.104	.287	.142	.003	.709	-.146	.288	.255	.006	.616
**PC (%) (R)**	.024	.013	3.623	.081	.064	**.030**	**.013**	**5.173**	**.115**	**.028**
**CV (mm/s) (R)**	.122	.175	.528	.013	.472	.150	.175	.704	.017	.406
**MCV (mm/s) (R)**	.537	.337	1.884	.040	.197	**.521**	**.338**	**3.976**	**.090**	**.049**
**LAT (s) (R)**	-.007	.010	0.502	.012	.483	.000	.010	.001	.000	.971
**DV (mm/s) (R)**	.058	.097	.451	.011	.506	.049	.981	.234	.006	.631
**MaxD (mm) (L)**	.175	.383	.213	.005	.647	.141	.384	.133	.003	.718
**MinD (mm) (L)**	.077	.246	.104	.003	.748	.002	.247	.000	.000	.992
**PC (%) (L)**	.004	.012	.136	.003	.714	.012	.012	1.752	.042	.193
**CV (mm/s) (L)**	.110	.197	.350	.008	.557	.091	.198	.225	.006	.638
**MCV (mm/s) (L)**	.306	.312	.896	.021	.349	.329	.312	1.116	.027	.297
**LAT (s) (L)**	-.012	.010	1.050	.025	.312	-.005	.010	.333	.008	.567
**DV (mm/s) (L)**	.204	.168	3.464	.084	.070	.112	.169	.885	.023	.353

*Coefficients presented in bold are significant (p < 0.05)

**TABLE 5B:** ANOVA results of the pupillometric changes between pre-game (GD-1) and post-game timepoints (GD+1 and GD+2)

ANOVA results	GD-1 to GD+1					GD-1 to GD+2				

	*Mean Difference*	*Std. Error*	*F*	η^2^	*p*	*Mean Difference*	*Std. Error*	*F*	η^2^	*p*
**MaxD (mm) (R)**	-.028	-.248	.013	.000	.910	-.000	.249	.000	.000	1.000
**MinD (mm) (R)**	-.004	.175	.001	.000	.981	-.046	.176	.066	.001	0.799
**PC (%) (R)**	-.004	.008	.380	.006	.540	.001	.008	.430	.001	0.836
**CV (mm/s) (R)**	-.029	.106	.076	.001	.784	-.001	.107	.000	.000	0.991
**MCV (mm/s) (R)**	.139	.205	.371	.005	.544	.123	.207	.498	.007	0.483
**LAT (s) (R)**	-.009	.006	1.810	.026	.183	-.001	.010	.065	.001	0.800
**DV (mm/s) (R)**	.037	.058	.435	.007	.512	.028	.059	.201	.003	0.656
**MaxD (mm) (L)**	-.007	.233	.001	.000	.975	-.042	.235	.032	.000	0.859
**MinD (mm) (L)**	.040	.150	.073	.001	.788	-.034	.151	.051	.001	0.822
**PC (%) (L)**	-.007	.007	.892	.013	.348	.000	.007	.004	.000	0.952
**CV (mm/s) (L)**	.052	.120	.179	.003	.674	.032	.121	.068	.001	0.796
**MCV (mm/s) (L)**	.104	.190	.298	.004	.587	.104	.190	.460	.007	0.500
**LAT (s) (L)**	.013	.006	3.819	.053	.055	**.020**	**.006**	**11.469**	**.146**	**0.001**
**DV (mm/s) (L)**	.075	.061	1.790	.027	.186	-.017	.061	.82	.001	0.776

*Coefficients presented in bold are significant (p < 0.05).

## DISCUSSION

The main purpose of this pilot study was to explore the potential usefulness of HQIPs in the context of monitoring game-induced fatigue in professional female basketball players. The reported findings may not only serve as a benchmark for future comparisons and hypothesis testing in athletic populations that includes PLR data from automated pupillometry, but also provide point estimates and variance for PLR measures, as well as inferential statistics to describe the effect of game-induced fatigue on pupillary behaviour, when used in naturalistic elite sports environment. Overall, the main findings of this pilot study suggest that (1) two out of seven pupillometrics represented good repeatability scores (MinD and MaxD) (ICC = 0.95–0.99), (2) Statistical significant relationships were found between MaxD, MinD, and all other biomarkers of game-induced fatigue (r = 0.69–0.82, p < 0.05), as well as between CV, MCV, and biomarkers of cognitive, lower-extremity muscle, and physiological game-induced fatigue (r = 0.74–0.76, p < 0.05), and (3) Statistically significant differences were found between rested and fatigued states for three pupillometrics: PC (right) and MCV (right), and LAT (left) (p < 0.05).

### The test-retest repeatability

In response to the first research question, good ICCs were reported for two out of seven pupillometrics, in particular: MinD (left) and MaxD (left and right) (0.95–0.99). Conversely, poor ICCs were reported for CV and PC (0.70–0.90) and very poor ICCs were reported for LAT, DV, and MCV (< 0.70). Nevertheless, the smallest read difference was extremely narrow for LAT in both eyes (0.005–0.007) as well as DV in both eyes (0.066–0.085). Therefore, the quantification of the maximum and minimum pupil diameter seem to be least prone to errors or noise due to external factors when examining professional female basketball players. However, this remains to be questioned as to the best of the authors knowledge, Swanson et al. (2017) [51] were the only researchers that provided open access to ICC results from PLR tests using the Neuroptics NPi-200 in an athletic population (i.e., 186 collegiate athletes across eight sports) [51]. Unfortunately, the only pupillometric reported in their investigation was the Neurological Pupil Index (NPI) (i.e., a proprietary score generated by the manufacturer). Furthermore, the PLR tests were completed at different time intervals, executed by multiple trained test administrators, and focused on a different use case (i.e., the detection of traumatic brain injury instead of fatigue monitoring). In turn, meta analyses and comparative inferences remain challenging. From a general viewpoint, the ICCs reported in this pilot study tend to follow the trend of various HQIPs applied in different use cases. For instance, Zheng et al. (2022) [52] also reported that LAT was the least reliable of all pupillometrics (i.e., very poor ICC of 0.65) using the RAPDx pupillometer (Konan Medical, Irvine, California, USA) and Chopra et al. (2020) [53] reported moderate to good ICCs for MinD and MaxD (ICC > 0.90) using the same RAPDx pupillometer.

Taking into account the abovementioned limitations, combined with the overall lack of consistency and transparency in pupillometric research over the past 50 years (as recently highlighted by an international panel of pupillometry experts across disciplines) [47], future researchers may use this pilot study as a baseline framework and prioritize transparency and standardization when executing their initiatives on this research topic.

### The relationship between pupillometrics and other biomarkers of game-induced fatigue

In response to the second research question, four pupillometrics were identified as the strongest indicators of game-induced fatigue in professional female basketball players. In particular, MaxD and MinD represented the strongest indicators for all other biomarkers of game-induced fatigue (r = 0.69–0.82, p < 0.05), whereas CV and MCV were identified as the strongest indicators for cognitive, lower-extremity muscle, and physiological biomarkers of game-induced fatigue (r = 0.74–0.76, p < 0.05). Hence, keeping track of these four pupillometrics on a daily basis may present a multi-modal solution to better understanding the psycho-physiological processes that underpin game-play fatigue in elite sports settings. However, the lack of existing literature on pupillometry in relation to sports-specific fatigue creates barriers for deeper comparative analyses. From a general perspective, the reported findings in this pilot study tend to align with previous investigations that examined the role of pupillometry in acute human fatigue. For instance, previous researchers have revealed strong relationships between multiple pupillometrics and biomarkers of HRV indices (e.g., lnRMSSD) [12, 14, 15, 54], as well as lower-extremity muscle fatigue (e.g. Postural Sway) [55, 56], subjective ratings of effort and tiredness from prolonged listening and attentional efforts) [57], subjective ratings of perceived exertion from muscular contraction during a power grip task [58]. Neverthelesss, there was a clear lack of consistency in terms of the selected testing timeframes (i.e., measuring before, during, or after given tasks or events), testing conditions (i.e., naturalistics vs. laboratory settings), selected HQIPs (i.e., self-engineered vs. commercial instruments), extracted pupillometrics (i.e., standard vs. proprietary scores and algorithms), and none of the investigations involved professional basketball competition. Acknowledging these limitations, and given that pupil responses vary based on the sport and context in which players participate in [kaltsatou, filipe], more detailed comparative analyses remain inappropriate at this point of time. Hence, a vigilant, transparent, and consistent research strategy is required to expand upon our existing knowledge regarding this use case.

### The time-course of pupillometrics from rested to fatigued states

In response to the third research question, three pupillometrics were capable of detecting a significant change from rested states (baseline and GD-1) to fatigued states (GD+1 and GD+2). In particular, PC (right) (F = 5.173, *η*^2^ = 0.115 p = 0.028) and MCV (right) (F = 3.976, *η*^2^ = 0.090 p = 0.049) significantly decreased from baseline to GD+2, while LAT (left) (F = 4.023, *η*^2^ = 0.109 p = 0.009) significantly increased from GD-1 to GD+2. Hence, at timepoints where residual fatigue was expected to remain present (48 h following games), the pupils constricted slower (MCV), with a smaller magnitude (PC), while it took longer to begin its constriction phase (LAT). This further supports the underlying physiological concept of pupillary behavior as LAT can be regarded as an index of sympathovagal balance (i.e., higher values indicate sympathetic dominance) [14], whereas PC and MCV can be regarded as an index of parasympathetic activity (i.e. higher values indicate parasympathetic dominance) [14]. Hence, this confirms, at least in part, that the players’ ANS were not fully reverted to normal levels 48-h following games. Interestingly, this trend of LAT, PC, and MCV is inconsistent with earlier findings by Kaltsatou et al. [14] who examined the immediate effects of physical exertion (maximal ergometer stress test) on pupillary behavior in power -and endurance-trained athletes. Specifically, in their investigation, LAT decreased, while MCV and PC increased from peak exertion to 5-min following the test (when heart rate return to baseline levels). Consequently, similar to how sports scientists typically evaluate traditional game-induced fatigue markers (e.g. Heart Rate Variability indices) [59, 60, 61], the before-after, day-to-day, and week-to-week fluctuations in pupillometics should be analyzed distinctively and individually, and contextualized against other external factors.

It is also important to acknowledge that the reported findings in this pilot study does not inform about the underlying factors that may have contributed to its overall acute fatigue state, nor does it imply the practical relevance of it. For instance, in a recent systematic review on post-game recovery kinetics in team ball sport athletes, Doeven et al. [62] highlighted the many covariables that play an influential role on the recovery dynamics of each player (e.g., menstrual cycle, physical fitness, role within the team, playing time, exertion, playing level, playing style, age, gender, genetic make-up, game location, preceding travel duration, opponent quality, imposed workload, lifestyle habits, sleep quantity and quality) [62]. Hence, future researchers are encouraged to integrate these cofactors in future investigations in order to pinpoint the underlying mechanisms for pupillary change following games. Additionally, to determine the practical relevance of these changes, future researchers may include predetermined anchor points that are practically relevant to their organization (e.g., specific injury occurrence per minute of activity exposure, on-court game-play performance metrics, pre-game alertness levels) [1, 59, 60]. This anchoring approach, often referred to as the Minium Clinical Important Difference (MCID), would allow practitioners to track pupillometrics per player over time and transform them into a prediction or prescription tool informing the onset to critical states via real-time alerting or traffic-light based visualization systems [59, 60, 61, 62]. For instance, Umesh et al. (2015) [63] were able to predict a self-reported Visual Analogue Scale (VAS) state of sleepiness score of ≥ 6 (the target variable) by using a MCV threshold value (age adjusted) of 2.8, with a sensitivity of 83% and specificity of 84%. Similarly, future researchers could determine the MC-ID’s for MaxD, MinD, CV, and MCV against their self-determined threshold values.

Finally, emerging technologies may enable faster interventions in the future. For instance, Stoeve et al. (2022) [64] created a VR-based stress test during a football goalkeeping scenario, and achieved a performance of 87.3% accuracy through the Random Forest classifier, claiming a comparable outcome to state-of-the-art approaches fusing eye tracking data and additional biosignals. Given the strong resurgence and further democratization of VR, Mixed Reality (MR) and augmented reality (AR) based eye-tracking applications in recent years [65–68], new opportunities may arise to accelerate pupillometric research in the context of real-time athlete monitoring.

In summary, the findings of this pilot study promotes HQIPs as a potential instrument for monitoring game-induced fatigue in female professional basketball players. From an ergonomic standpoint, the PLR testing routine took little time and effort on the practitioner’s side, and good test-retest repeatability scores were reported for two pupillometrics (MaxD and MinD). Additionally, strong relationships were found for four pupillometrics (MaxD, MinD, CV, and MCV) and all other biomarkers of game-induced fatigue, and three pupillometrics were able to distinguish rested states from fatigued states (LAT, PC, and MCV). Although these preliminary findings tend to support the potential adoption of pupillometry as an athlete monitoring tool in elite sports settings, researchers should remain cautious when drawing conclusive inferences as the dataset was extracted from a relatively small and homogenous sample, tracked over a relatively short timeframe (4 games across 5 weeks). Therefore, future researchers should aim to cover a larger and more heterogenous sample across various time intervals to allow for more precise estimations of “normal pupillary behaviour” in elite athletes. The recent technological advancements in HQIPs that are compact and versatile (e.g., smartphone-based and VR-based pupillometers) [63–70] may further accelerate and facilitate investigations on this topic.

## CONCLUSIONS

HQIPs have opened a new window of opportunities for sports practitioners given its ease of use and ability to extract objective insights on player fatigue in a quick, reliable, valid, and non-invasive character. Overall, the pupillometrics MinD, MaxD, CV, and MCV were identified as the most promising indicators of game-induced fatigue in female professional basketball players. However, it’s important to acknowledge that this research line is still in its infancy, and the findings stem from a small homogenous sample, thus the statistical inferences remain indicative rather than confirmative or directive. However, future researchers are encouraged to leverage this pilot study as a baseline framework for future investigations, and ensure standardization is prioritized throughout the process in order to maximize the reproducibility of findings across a variety of sports, timeframes, contexts, and use cases.
